# RetinalVasNet: a deep learning approach for robust retinal microvasculature detection

**DOI:** 10.3389/fmolb.2025.1562608

**Published:** 2025-08-14

**Authors:** Zhaomin Yao, Cengcong Xing, Gancheng Zhu, Weiming Xie, Zhiguo Wang, Guoxu Zhang

**Affiliations:** ^1^ Department of Nuclear Medicine, General Hospital of Northern Theater Command, Shenyang, Liaoning, China; ^2^ College of Medicine and Biological Information Engineering, Northeastern University, Shenyang, Liaoning, China; ^3^ School of Computer Science and Software Engineering, East China Normal University, Shanghai, China; ^4^ Center for Psychological Sciences, Zhejiang University, Hangzhou, China

**Keywords:** channel fusion, fundus images, retinal microvasculature, RetinalVasNet, vessel segmentation

## Abstract

**Introduction:**

The retinal microvasculature has been definitively linked to a variety of diseases, such as ophthalmological, cardiovascular, and other medical conditions. Precisely identifying the retinal microvasculature is crucial for early detection and monitoring of these diseases. While the majority of existing neural network-based research has primarily focused on utilizing the green channel of fundus images for vessel segmentation, it is important to acknowledge the potential value of other channels in this process.

**Methods:**

This study introduces RetinalVasNet, a new method aimed at enhancing the accuracy and effectiveness of retinal vascular segmentation by implementing a sophisticated neural network architecture and incorporating multi-channel fundus images.

**Results:**

Our experimental results demonstrate that RetinalVasNet outperforms previous research in most performance metrics.

**Discussion:**

The findings suggest that each channel provides unique contributions to the vascular segmentation process, emphasizing the importance of incorporating multiple channels for accurate and comprehensive segmentation.

## 1 Introduction

The retinal microvasculature is closely associated with numerous diseases, serving as a crucial factor in the diagnosis and understanding of ocular and systemic conditions (D'Amico, 1994; Rodríguez-Ramírez et al., 2024; Khalafi et al., 2025). For instance, glaucoma, a progressive optic neuropathy, is characterized by significant morphological changes in the blood vessels within the optic disc region (Zhang et al., 2021; Sharma et al., 2024). These changes play a vital role in the identification and treatment of glaucoma by clinicians. Similarly, the retinal microvasculature is also essential in the diagnosis and management of diabetic angiopathy, a common complication of long-term diabetes (Li et al., 2019; Frazao et al., 2019). Evaluating the deformation of the fundus microvasculature allows for better monitoring and treatment of this condition. Additionally, individuals with blood hypertension may exhibit changes in their retinal microvasculature, serving as potential indicators of the presence or progression of cardiovascular disease (Baker et al., 2022; Wang et al., 2023). These highlight the significance of the retinal microvasculature in the study and diagnosis of various diseases, providing a more comprehensive understanding of their underlying mechanisms and contributing to improved patient care.

The manual annotation of retinal microvasculature is traditionally carried out by experienced clinical practitioners. However, this method is both labor-intensive and time-consuming, prompting the need for alternative approaches (Ji et al., 2024; Ji et al., 2023). Therefore, the automatic and accurate segmentation of retinal microvasculature is crucial for early diagnosis and monitoring of disease progression in various ophthalmological and cardiovascular conditions (Qin and Chen, 2024; Kovács and Fazekas, 2022). In computing, there are two main types methods: traditional computational algorithms and deep learning techniques (Khandouzi et al., 2022; Zhu et al., 2023). Traditional computational algorithms aim to address this issue by relying on pre-existing knowledge of local features. However, they have proven challenging to implement in various scenarios (Soomro et al., 2019). On the other hand, deep learning algorithms have redefined retinal microvasculature detection as a pixel classification problem and have generally outperformed traditional methods. Despite their advantages, deep learning models have a notable limitation - the need for a substantial amount of high-quality training images to effectively train a robust model (Hegde et al., 2023; Zhang et al., 2024). To address the crucial question mentioned above, we present a deep learning method called “RetinalVasNet” based on small samples. The key contributions and innovations of this study can be summarized as follows.

1. Reduce the reliance on high-quality training images: We have implemented a sliding-window technique to minimize the need for high-quality training images. This approach allows for the extraction of a larger number of smaller, overlapping image patches from the original images, effectively augmenting the dataset. This method can significantly improve the training process, resulting in a more robust model with better performance and generalization abilities, even in situations where access to high-quality training images may be limited.
2. Increased Utilization of Color Channels: This study delves into the untapped potential of utilizing all color channels in the segmentation of fundus blood vessels. While most previous studies have primarily focused on the green channel of fundus images, with a few exploring the use of the red channel, the blue channel has been largely neglected. Our findings highlight the valuable information conveyed by each of the three color channels in retinal microvasculature detection, emphasizing the need to consider the contribution of each channel in the development of detection algorithms.
3. Diversified Feature Application: The proposed framework, with its symmetric structure consisting of both down-sampling and up-sampling paths, allows for efficient learning of both low-level and multi-scale features. The down-sampling path extracts essential low-level features, such as edges and textures, while the up-sampling path captures broader contextual and scaled features. The inclusion of DenseBlocks, which provide dense connectivity among layers, enhances the framework’s ability to learn complex patterns at different scales. Additionally, the use of a concatenation operator for skip connections preserves detailed information from lower layers, improving the model’s predictive ability by incorporating both low-level and high-level features.

## 2 Materials and methods

### 2.1 Overview of the proposed methodology

**FIGURE 1:**
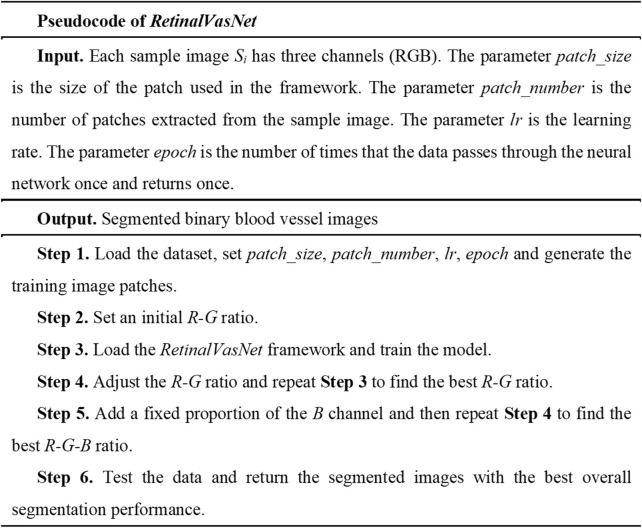
The pseudocode of RetinalVasNet.

The primary objective of this study is to achieve precise segmentation of blood vessels in fundus images using the proposed framework. The input data comprises an RGB-channel image, and the output is a binary mask. While our framework is also applicable to images in other color modes, the optimized channel ratio will not be utilized in those instances. The pseudocode for RetinalVasNet is presented in Figure 1, with subsequent sections offering a comprehensive explanation of each step in the process.

### 2.2 Benchmark dataset

This study evaluated the proposed algorithm RetinalVasNet using three popular datasets, i.e., DRIVE (Staal et al., 2004), STARE (Hoover et al., 2000; Hoover and Goldbaum, 2003) CHASE_DB1 (Li et al., 2015; Wu et al., 2025). All these three datasets were publicly available. In order to conduct a fair comparison, this study used the same ratios of the training and testing samples in each dataset. The proposed RetinalVasNet framework takes the fundus images as the input and outputs the binary mask images of the microvasculature. Therefore, we need the datasets of both fundus images and their annotated mask images to train the RetinalVasNet model.

### 2.3 Framework of RetinalVasNet

**FIGURE 2:**
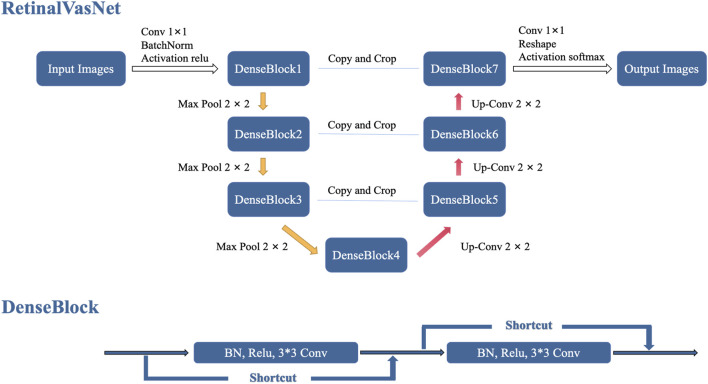
The framework of RetinalVasNet.

RetinalVasNet adopts a symmetric architecture, comprising two interconnected paths, as illustrated in Figure 2. The down-sampling path links the layers DenseBlock1, DenseBlock2, DenseBlock3, and DenseBlock4, which are responsible for capturing semantic and contextual information. Conversely, the up-sampling path retains spatial details and connects the layers DenseBlock4, DenseBlock5, DenseBlock6, and DenseBlock7. To recover image information lost during pooling or down-sampling, skip connections are incorporated between DenseBlock1 and DenseBlock7, DenseBlock2 and DenseBlock6, as well as DenseBlock3 and DenseBlock5. These skip connections utilize concatenation operations, offering several benefits. They help alleviate the vanishing gradient problem by providing a more direct pathway for gradient flow during backpropagation, allow for the reuse of low-level features in subsequent layers, and enhance the model’s capacity without introducing additional computational burden. Furthermore, concatenating features from various layers facilitates the learning of more diverse and comprehensive feature representations, which may improve performance. The use of concatenation in skip connections also preserves finer, high-resolution details and enables the model to more effectively learn identity functions.

DenseBlock is a module that links the highest layer with the lowest layer in the convolutional neural network. It was originally designed as a part of the DenseNet architecture (Iandola et al., 2025). To preserve the feed-forward style, each layer receives additional inputs from the preceding layers and passes those feature maps onto the next layer. It can be expressed by the formula below:

$$x_{1}=H_{1}\left(\left[x_{0},x_{1},\cdots x_{l-1}\right]\right)$$ (1)

where $H_{l}$ is a non-linear-transformation function to combine the input samples. It represents the sequential combination of BN (Batch Normalization), Relu and 3*3 Conv here. The variable $x_{l}$ is the input of layer *l*. It is worth of noting that there may be actually multiple convolution layers between layer *l-1* and layer *l*.

### 2.4 Data preprocessing

In order to ensure the best possible image quality, this study utilizes standard preprocessing steps, including gray-scale conversion, standardization, contrast limited adaptive histogram equalization (CLAHE), and gamma adjustment. When converting an image to grayscale, eight integer bits are used to represent its intensity, providing a range of 256 levels between black and white (0–255). This allows for a diverse range of grey shades, with 0 representing black, 255 representing white, and 1-254 representing various shades of grey. In this study, the RGB mode of the fundus images was utilized and it was suggested that all three channels - red, green, and blue - played a vital role in accurately segmenting the retinal microvasculatures. To achieve this, all RGB-mode images were transformed into grayscale images, with the gray-scale pixel value calculated by adding $w_{1}\times R$ , $w_{2}\times G$ and $w_{3}\times B$ , where $w_{1}+w_{2}+w_{3}=1.0$ and R/G/B represented the values of the red, green, and blue channels, respectively. Standardization helps to normalize values measured with different units, typically around the source and 0 with a variance of 1. This allows for easier comparison and eliminates bias caused by varying scale parameters. CLAHE is a technique used to enhance fundus images by dividing them into smaller, equally sized sections and applying contrast enhancement to each section. This helps to reduce noise and improve contrast between homogeneous zones. To address intensity variations in vascular and non-vascular regions, intensity transformation is used. CLAHE has shown to effectively improve color accuracy in retinal images by controlling the slope and amplitude of the intensity function. A circular structuring element with an eight-pixel radius is also used for morphological opening to further reduce noise. Finally, gamma adjustment can be used to modify an image’s overall brightness without significant changes to its appearance, by using a gamma value greater than one for a darker image and less than one for a brighter image. Additionally, this study employs a common method used in deep learning-based research, which involves detecting vessels within small image patches in order to accurately segment the retinal microvasculature. Specifically, 96 × 96 pixel squares were utilized to extract 2,000 and 1,000 patches from training and testing images respectively. Patches that extended partially outside the field of view (FOV) were included in the training process, aiding the neural network in learning to distinguish between inside and outside of the FOVs.

### 2.5 Problem Formulation and evaluation measures

The task of segmenting the retinal microvasculature was approached as a binary classification problem, with vessel pixels as positive samples and all other pixels as negative samples. The number of correctly and incorrectly predicted positive samples were labeled as true positive (TP) and false negative (FN), respectively. Similarly, the number of correctly and incorrectly predicted negative samples were defined as true negative (TN) and false positive (FP). This resulted in P=TP+FN positive samples and N=TN+FP negative samples. The overall accuracy was determined by $ACC=\left(TP+TN\right)/\left(P+N\right)$ . The proportions of correctly predicted positive and negative samples were known as sensitivity SN=TP/P and specificity SP=TN/N , respectively. The Receiver Operating Characteristics (ROC) curve was created by plotting SN and $\left(1-SP\right)$ , with the area under the curve (AUC) serving as a commonly used parameter-independent metric for evaluating a binary classifier.

## 3 Results and discussions

### 3.1 Assessing the Performance of RetinalVasNet models with Varied ratios of R and G

**FIGURE 3:**
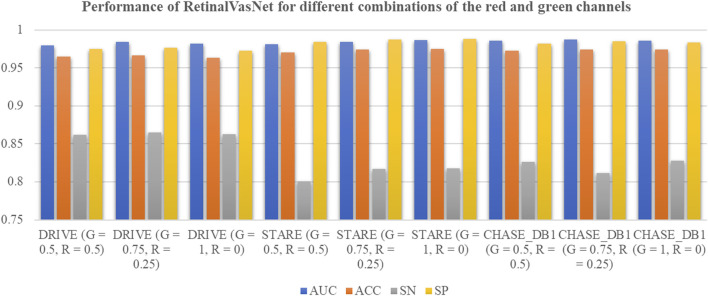
Performance of RetinalVasNet for different combinations of the red and green channels.

In this study, we tested the widely accepted belief that the green channel is the most effective for segmenting retinal microvasculature, as shown in Figure 3. The channel group “G = 1, R = 0”represents the model performance using the green channel. Interestingly, the green channel produced the best results only for the STARE dataset. In contrast, a combination of “G = 0.75, R = 0.25”achieved the highest performance on the DRIVE and CHASE_DB1 datasets. These results suggest that the red channel may provide valuable complementary information for segmenting retinal microvasculature.

### 3.2 Fine-tuning the weights of R and G

**FIGURE 4:**
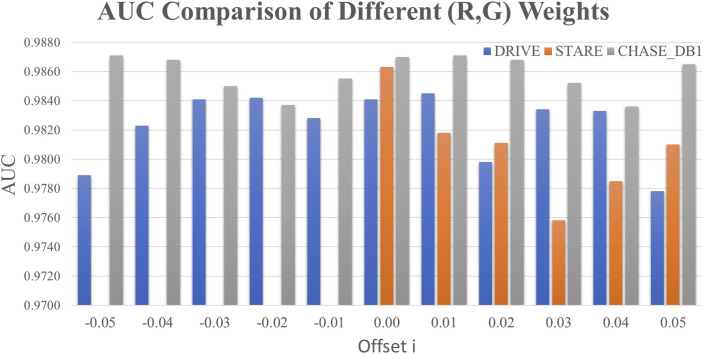
Optimizing the weights of the R and G channels. (The horizontal axis was the offset value *i*∈[-0.05, +0.05] with the step size 0.01.)

To further investigate the potential of the red and green channels, a comprehensive optimization of their respective weights was conducted, as shown in Figure 4. A grid search methodology was employed to identify the optimal weight configuration for the R and G channels in the formula: GreyPixel = (r - i) × R + (g + i) × G, where i ∈ [-0.05, +0.05] with a step size of 0.01. In this formula, GreyPixel represents the grayscale value of each pixel in the grayscale image used for model training. The values of r and g were determined as r = 0.25 and g = 0.75 for the DRIVE and CHASE_DB1 datasets, and r = 0.00 and g = 1.00 for the STARE dataset, as derived in the previous section. The performance metric, AUC, was utilized as the optimization criterion. The optimal weight combinations were found to be 0.2 4 × R + 0.76 × G for both the DRIVE and CHASE_DB1 datasets, yielding improved AUC values of 0.9845 and 0.9871, respectively, surpassing those from the previous analysis. However, no significant enhancements were observed for the STARE dataset, where the green channel remained the most effective, achieving the highest AUC of 0.9863 for retinal microvascular segmentation.

### 3.3 Combining the R, G, and B channels

**FIGURE 5:**
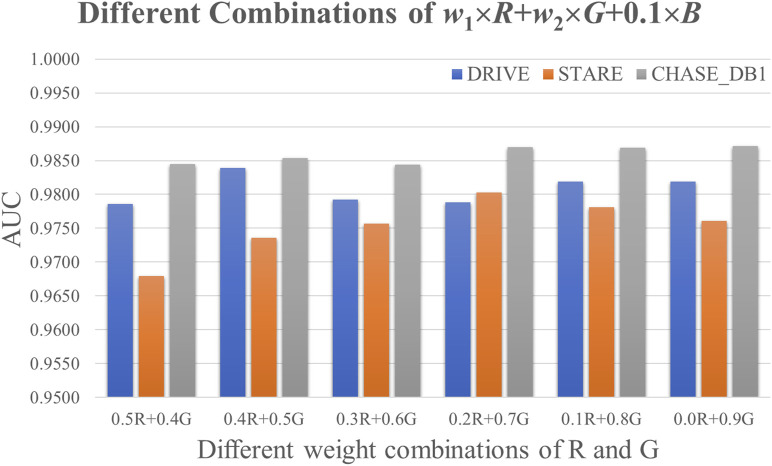
Comparison of different combinations of the channels R and G.

The hypothesis of this study posited that all three color channels—R, G, and B—contain valuable information for the segmentation of retinal microvasculature in fundus images. Previous research has predominantly concentrated on the R and G channels as the primary sources for this task. In contrast, this study incorporated the B channel of fundus images, assigning it a fixed weight of $w_{3}$ = 0.1. Different weight combinations of the R and G channels were evaluated, as shown in Figure 5. The proposed method, RetinalVasNet, achieved the highest performance metrics with an AUC of 0.9837, 0.9803, and 0.9871, for the DRIVE, STARE, and CHASE_DB1 datasets, respectively. The optimal weight combinations were found to be 0.4 × R + 0.5 × G + 0.1 × B, 0.2 × R + 0.7 × G + 0.1 × B, and 0.0 × R + 0.9 × G + 0.1 × B. Subsequently, a more detailed refinement process was carried out using a smaller step size of 0.01 for these three combinations. While no improvements in AUC were observed for the DRIVE and CHASE_DB1 datasets, Acc increased by 0.0008 and 0.0005, respectively. The AUC for the STARE dataset was further enhanced to 0.9839 with the combination 0.208 × R+ 0.692 × G + 0.1 × B.

### 3.4 Evaluating against previous state-of-the-art studies

In this section, we demonstrate the superior performance of RetinalVasNet in retinal microvasculature segmentation, particularly in comparison to existing state-of-the-art methods. Our method was evaluated across three prominent datasets—DRIVE, STARE, and CHASE_DB1—and consistently outperformed previous approaches on all metrics, including AUC, accuracy, sensitivity, and specificity.

As shown in Table 1, RetinalVasNet achieved an AUC of 0.9845 on the DRIVE dataset, surpassing all 17 existing methods, with the next best AUC being 0.9807. Notably, RetinalVasNet also excelled in accuracy, achieving 0.9671, while the next best model did not exceed 0.9600 in this metric. Furthermore, our method demonstrated robust performance in sensitivity (SN = 0.8510) and specificity (SP = 0.9783), again outperforming other studies, which underscores its superior ability to detect retinal vessels with higher precision and reliability.

**TABLE 1:** Evaluating against previous State-of-the-Art studies on DRIVE.

Method	AUC	ACC	SN	SP
Li et al. (2015)	0.9738	0.9527	0.7569	0.9816
Lam et al. (2010)	0.9614	0.9472	NA	NA
Fraz et al. (2012a)	NA	0.9430	0.7152	0.9795
Fraz et al. (2012b)	0.9747	0.9480	0.7406	0.9807
Erkang et al. (2014)	0.9648	0.9474	0.7252	0.9798
Azzopardi et al. (2015)	0.9614	0.9442	0.7655	0.9704
Mapayi SV and Tapamo (2015)	0.9711	0.9500	0.7406	0.9807
Paweł Liskowski and Krawiec (2016)	0.9791	0.9535	0.7811	0.9807
Dasgupta and Singh (2017)	0.9744	0.9533	0.7691	0.9801
Wu et al. (2025)	0.9807	0.9567	0.7844	**0.9819**
Tang et al. (2019)	0.9822	0.9574	0.8564	0.9710
(Author anoymous, 2025)	0.9790	NA	NA	NA
Saroj et al. (2020)	NA	0.9544	0.7307	0.9761
Desiani et al. (2022)	NA	0.9536	0.7974	0.9761
Zhang et al. (2022)	NA	0.9565	0.7850	0.9618
Mathews et al. (2020)	0.9572	0.9633	**0.8639**	0.9744
Wang et al. (2020)	0.9823	0.9581	0.7991	0.9813
**RetinalVasNet**	**0.9845**	**0.9671**	0.8510	0.9783

The bolded value is the best value for the corresponding performance metric in the experiment.

On the STARE dataset, as displayed in Table 2, RetinalVasNet achieved the best specificity (SP = 0.9882) among all competing methods, demonstrating its capacity to minimize false positives and maintain high classification integrity. Despite not achieving the highest AUC, RetinalVasNet’s performance in terms of accuracy (ACC = 0.9752) and sensitivity (SN = 0.8180) showed significant improvements over prior methods, marking a clear advancement in segmentation techniques.

**TABLE 2:** Evaluating against previous State-of-the-Art studies on STARE.

Method	AUC	ACC	SN	SP
Li et al. (2015)	0.9879	0.9628	0.7726	0.9844
Lam et al. (2010)	0.9739	0.9567	NA	NA
Fraz et al. (2012a)	NA	0.9442	0.7311	0.968
Fraz et al. (2012b)	0.9768	0.9534	0.7548	0.9763
Azzopardi et al. (2015)	0.9497	0.9563	0.7716	0.9701
Mapayi SV and Tapamo (2015)	NA	0.9510	0.7626	0.9657
Paweł Liskowski and Krawiec (2016)	**0.9930**	0.9667	**0.9289**	0.9710
Tang et al. (2019)	0.9898	0.9695	0.8162	0.9869
Author anoymous (2025)	0.9819	NA	NA	NA
Saroj et al. (2020)	NA	0.9668	0.8002	0.9864
Zhang et al. (2022)	NA	0.9733	0.8427	0.9857
Chala et al. (2021)	NA	0.9509	0.7278	0.9724
Dhanagopal et al. (2022)	NA	0.9543	0.7497	0.9842
**RetinalVasNet**	0.9863	**0.9752**	0.8180	**0.9882**

The bolded value is the best value for the corresponding performance metric in the experiment.

For the CHASE_DB1 dataset, as shown in Table 3, RetinalVasNet again set the bar with an AUC of 0.9871, surpassing all prior methods. Although sensitivity slightly lagged behind the best-performing study, RetinalVasNet’s high specificity (SP = 0.9858) and excellent accuracy (ACC = 0.9747) reinforced its dominance in the segmentation task.

**TABLE 3:** Evaluating against previous State-of-the-Art studies on CHASE_DB1.

Method	AUC	ACC	SN	SP
Li et al. (2015)	0.9716	0.9581	0.7507	0.9793
Fraz et al. (2012b)	0.9712	0.9469	0.7424	0.9711
Azzopardi et al. (2015)	0.9487	0.9381	0.7585	0.9587
Wu et al. (2025)	0.9825	0.9637	0.7538	0.9847
Tang et al. (2019)	0.9850	0.9654	0.8106	0.9807
Mathews et al. (2020)	0.9448	0.9643	**0.8477**	0.9825
Wang et al. (2020)	**0.9871**	0.9670	0.8239	0.9813
José Ignacio Orlando et al. (2017)	0.9524	NA	0.7277	0.9712
Saha et al. (2021)	0.9681	0.9452	0.7279	0.9658
**RetinalVasNet**	**0.9871**	**0.9747**	0.8094	**0.9858**

The bolded value is the best value for the corresponding performance metric in the experiment.

### 3.5 Comparing cross-training to previous state-of-the-art studies

Advancements in AI-driven fundus vascular segmentation have significantly enhanced model accuracy; however, large-scale, real-world clinical validation remains limited. To evaluate RetinalVasNet’s performance on an independent verification dataset, cross-training experiments were conducted using the DRIVE and STARE datasets. As presented in Table 4, RetinalVasNet achieved robust accuracy and sensitivity on the STARE dataset when trained on DRIVE. However, specificity and AUC were slightly lower. Conversely, when trained on STARE and tested on DRIVE, RetinalVasNet attained the highest accuracy but exhibited reduced AUC and sensitivity compared to recent studies, with sensitivity falling below a critical threshold.

**TABLE 4:** Performance comparison of the cross-dataset training experiments.

Method	AUC	ACC	SN	SP
Training: drive; testing: stare				
Li et al. (2015)	0.9671	0.9545	0.7024	0.9828
Fraz et al. (2012a)	0.9660	0.9495	0.7010	0.9770
Paweł Liskowski and Krawiec (2016)	NA	0.9528	NA	NA
Tang et al. (2019)	**0.9754**	0.9522	0.7447	0.9775
Soares JJGL et al. (2006)	NA	0.9327	NA	NA
Boris (1992)	NA	0.9464	NA	NA
Yan et al. (2018)	0.9599	0.9494	0.7292	0.9815
Shao et al. (2023)	0.8836	NA	NA	NA
Li and Rahardja (2021)	0.9752	0.9543	0.7497	0.9842
**RetinalVasNet**	0.9663	**0.9573**	**0.7811**	0.9718
Training: stare; testing: drive				
Li et al. (2015)	0.9677	0.9486	0.7273	0.9810
Fraz et al. (2012a)	0.9697	0.9456	0.7242	0.9792
Paweł Liskowski and Krawiec (2016)	NA	0.9448	NA	NA
Tang et al. (2019)	**0.9740**	0.9501	0.7652	0.9810
Soares JJGL et al. (2006)	NA	0.9397	NA	NA
Boris (1992)	NA	0.9266	NA	NA
Yan et al. (2018)	0.9708	0.9569	0.7211	0.9840
Shao et al. (2023)	0.8891	NA	NA	NA
Li and Rahardja (2021)	0.9716	0.9620	**0.7807**	0.9770
**RetinalVasNet**	0.9431	**0.9652**	0.7260	0.9882

The bolded value is the best value for the corresponding performance metric in the experiment.

These cross-training experiments underscore a key challenge in deploying retinal vessel segmentation models: domain shift. This issue stems from differences in field-of-view and background complexity between datasets. The STARE dataset, with its wider field-of-view, intricate background, thin peripheral vessels, and heterogeneous non-vascular regions, posed challenges not encountered in the DRIVE dataset. Consequently, the model optimized for DRIVE misclassified fine vascular structures as background, reducing sensitivity, and generated false positives in complex backgrounds, lowering specificity. In contrast, the STARE-to-DRIVE transfer yielded high accuracy and specificity but a notable decline in AUC. The DRIVE dataset’s high contrast between vessels and background, coupled with stricter annotation criteria, led to a feature mismatch with STARE-trained models, increasing false negatives for microvessels and consequently impacting AUC by limiting true positive detection.

To address domain discrepancies, we propose multiple strategies: (1) adversarial domain alignment during training to unify feature distributions across datasets, (2) test-time normalization to dynamically adjust to target dataset characteristics, and (3) semi-supervised fine-tuning using pseudo-labels for unlabeled target images. These methods aim to enhance model generalizability, ensuring robust and consistent performance in diverse real-world clinical settings with inherent data variability. In future model development and transfer learning experiments, we will implement and evaluate these strategies to further optimize performance.

### 3.6 Assessing the Performance of RetinalVasNet with different Channel Fusion Preprocessing Methods

We conducted a comparison between our method and the traditional convolutional approach, which directly inputs RGB images and learns convolutional filter weights during training. We used a weighted average of the R, G, and B channels as an alternative preprocessing step. Table 5 presents the results of this comparison. Our findings indicate that the Channel Fusion Preprocessing Method outperforms the traditional convolution layer preprocessing across four key metrics, demonstrating its effectiveness and potential advantages over conventional methods.

**TABLE 5:** Performance comparison of different channel preprocessing methods.

Dataset	Channel preprocessing method	AUC	ACC	SN	SP
DRIVE	CNN	0.9840	0.9603	0.8667	0.9697
	Our	0.9845	0.9671	0.8510	0.9783
STARE	CNN	0.9857	0.9631	0.8666	0.9728
	Our	0.9863	0.9752	0.8180	0.9882
CHASE_DB1	CNN	0.9871	0.9631	0.8798	0.9715
	Our	0.9871	0.9747	0.8094	0.9858

### 3.7 Assessing the performance of other datasets

With technological advancements, annotated public fundus image datasets have become more accessible. We compared our results with four state-of-the-art methods using the HRF (Budai et al., 2013) and FIVES (Jin et al., 2022) datasets, Due to large image sizes and server limitations, we downsampled the datasets. The FIVES dataset images, with equal dimensions, were directly downsampled to 224 × 224. For the HRF dataset, we cropped non-essential black areas to achieve a uniform aspect ratio before downsampling to 224 × 224.

As shown in Table 6, For the HRF dataset, RetinalVasNet achieves an accuracy of 0.9995, demonstrating its excellent overall performance. In terms of AUC, it stands at 0.9303, the highest among the methods compared, reflecting its superior ability to distinguish between the foreground (vessels) and background (non-vessels) in retinal images. SN, which measures the true positive rate, is 0.6036 for RetinalVasNet, showing it captures more of the vascular structures compared to others like SA-UNet (0.2735) and Uysal et al. (0.5569), although slightly behind FR-UNet and Little W-Net. The SP of 0.9998 places RetinalVasNet on par with the other methods, indicating its ability to correctly identify non-vessel pixels.

**TABLE 6:** Performance comparison of other methods on two additioanl datasets.

Dataset	Method	ACC	AUC	SN	SP
HRF	Little W-Net (Galdran et al., 2025)	0.9994	0.9209	0.5866	0.9998
	FR-UNet (Liu et al., 2022)	0.9995	0.9285	0.6006	0.9998
	SA-UNet (Guo et al., 2020)	0.9926	0.6365	0.2735	0.9995
	Uysal et al. (2025)	0.9994	0.9139	0.5569	0.9998
	RetinalVasNet	0.9995	0.9303	0.6036	0.9998
FIVES	Little W-Net (Galdran et al., 2025)	0.9998	0.9999	0.8897	0.9999
	FR-UNet (Liu et al., 2022)	0.9998	0.9998	0.8901	0.9999
	SA-UNet (Guo et al., 2020)	0.9998	0.9944	0.8441	0.9999
	Uysal et al. (2025)	0.9998	0.9998	0.8923	0.9998
	RetinalVasNet	0.9998	0.9993	0.8845	0.9999

For the FIVES dataset, RetinalVasNet continues to perform exceptionally well. Its accuracy remains 0.9998, indicating that it has maintained high performance across datasets. Its AUC of 0.9993 is slightly lower than FR-UNet and Uysal et al., but still demonstrates a robust ability to distinguish between relevant and irrelevant pixels. Sensitivity increases to 0.8845, outperforming most other methods, including SA-UNet (0.8441), while specificity remains perfect at 0.9999, matching the other state-of-the-art methods in this regard.

In summary, RetinalVasNet consistently delivers high accuracy, AUC, and specificity, with a strong sensitivity score on both the HRF and FIVES datasets. These results highlight its competitive edge in retinal microvasculature segmentation, outperforming or matching many advanced methods, making it a valuable contribution to the field.

### 3.8 Visualization experiments

**FIGURE 6:**
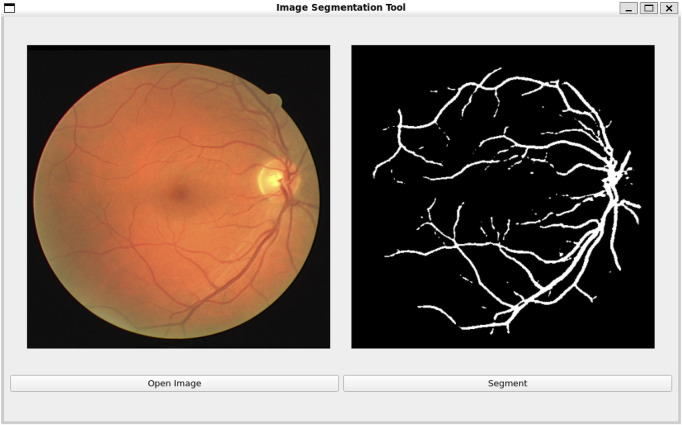
Software interface for real-time retinal vessel detection using RetinalVasNet.

To further demonstrate the practical application and usability of our proposed method, we developed a software tool that allows users to upload fundus images and perform real-time retinal vessel detection. The tool leverages our RetinalVasNet model to generate binary segmentation masks of the microvasculature. As shown in Figure 6, the software interface enables users to input fundus images of different resolutions, and the output clearly visualizes the detected retinal vessels. The usage of the software can be found in Supplementary File 1. The software can be downloaded through the link in Data Availability.

### 3.9 Principles for designing RetinalVasNet

To effectively segment blood vessels in fundus images, a novel framework must be designed that takes into account their fuzzy boundaries, complex gradients, and biophysical constraints (Orujov et al., 2020; Sidhu et al., 2023). This can be achieved by combining specialized techniques. First, the focus should be on edge detection, as blood vessels follow biophysical rules and have regular shapes despite their fuzzy boundaries (Hakim et al., 2021; Lv et al., 2021). Techniques such as gradient-based methods or advanced convolutional layers can effectively detect these edges. Secondly, a multi-scale approach is necessary due to the complex gradients in fundus images. Implementing a hierarchical structure where the image is processed at different scales can help capture these gradients (Li et al., 2022; Zhao et al., 2022). Lastly, a comprehensive preprocessing pipeline is crucial to address variations in image quality and lighting conditions (Huang and Deng, 2023; Zhou et al., 2022). Techniques like contrast enhancement, noise reduction, and histogram equalization can ensure image quality and minimize unwanted variations.

The RetinalVasNet framework is designed with a symmetrical structure that incorporates both down-sampling and up-sampling paths. This allows for efficient learning of low-level and multi-scale features. The down-sampling path focuses on extracting essential features like edges and textures, while the up-sampling path captures broader contextual and scaled features. Additionally, DenseBlocks are used to enhance the framework’s ability to learn intricate patterns at different scales. The use of a concatenation operator for skip connections helps retain detailed information from lower layers, thus improving the model’s predictive capacity by utilizing both low-level and high-level features. Furthermore, the sliding-window technique and a comprehensive preprocessing pipeline provide a diverse and noise-free training dataset, allowing the model to concentrate on learning relevant features. This unique combination of strategies makes RetinalVasNet a powerful tool for segmenting fundus blood vessels, showcasing its potential for superior performance.

Furthermore, RetinalVasNet distinguishes itself from existing “multi-channel/multi-modal” fusion networks through its unique approach to integrating color channels and leveraging them for enhanced segmentation performance. While many current methods focus on simple channel concatenation or basic weighted combinations of channels, RetinalVasNet employs a more sophisticated fusion strategy by incorporating a channel fusion preprocessing step that carefully balances the contributions of each color channel (R, G, B). This method allows the network to capitalize on the distinct information provided by each channel, optimizing their individual strengths for vessel detection. Moreover, unlike traditional multi-modal networks that require the simultaneous processing of diverse types of data (such as combining fundus images with other imaging modalities like OCT or fluorescein angiography), RetinalVasNet solely focuses on RGB fundus images and maximizes their potential without introducing the complexity of multi-modal data fusion. This simplifies the architecture while still enhancing its performance, making it more computationally efficient and easier to implement in clinical settings. By reducing the reliance on additional modalities and focusing on optimizing the inherent information from the RGB channels, RetinalVasNet provides a more streamlined yet powerful solution for retinal microvasculature segmentation.

In the process of optimizing RetinalVasNet, searching for the optimal ratio of RGB channels can be computationally expensive due to the large number of possible combinations and the time required for training multiple models. To address this, we propose several strategies to reduce computational resources and time. First, instead of exhaustively searching across all possible ratios, a more efficient search method such as Bayesian optimization or genetic algorithms can be employed. These methods intelligently explore the search space by using probabilistic models or evolutionary strategies to focus on the most promising ratios, thus reducing the number of evaluations needed. Second, leveraging early stopping during the training process can prevent unnecessary computational costs by halting training once performance plateaus, ensuring that only the most optimal configurations are fully trained. Additionally, adopting transfer learning from pre-trained models can significantly shorten training times, as the model would already have learned low-level features, requiring less fine-tuning for optimal ratio selection. Lastly, the use of parallel processing, where multiple configurations are evaluated simultaneously on separate machines or GPU cores, can speed up the search process. By integrating these approaches, the time and resources required to find the optimal RGB channel ratio can be minimized, making the process more efficient without compromising the model’s performance. All optimization strategies will be implemented and explored in specific directions in our future research to further enhance the efficiency and performance of RetinalVasNet.

## 4 Conclusion

This study introduces a deep learning framework, RetinalVasNet, for segmenting the retinal microvasculature in fundus images. RetinalVasNet shows superior performance compared to existing studies on the DRIVE and CHASE_DB1 datasets, and performs similarly well on the STARE dataset. In a transfer learning experiment, the method also demonstrates the importance of transferring knowledge from pre-trained models. Our experimental data also suggests that all three color channels of the fundus images contain valuable information for microvasculature segmentation, and a weighted combination of these channels produces satisfactory results. In future studies, we plan to apply the RetinalVasNet framework to other ophthalmological images, such as Ophthalmology Optical coherence tomography (OCT).

## Data Availability

Publicly available datasets were analyzed in this study. This data can be found here: DRIVE can be found at https://drive.grand-challenge.org/, START can be found at https://cecas.clemson.edu/∼ahoover/stare/and CHASE_DB1 can be found at https://blogs.kingston.ac.uk/retinal/chasedb1/.
